# Finite Element Analysis of a Rib Cage Model: Influence of Four Variables on Fatigue Life during Simulated Manual CPR

**DOI:** 10.3390/bioengineering11050491

**Published:** 2024-05-15

**Authors:** Jong Hyeok Jeon, Jae Ho Sul, Dae Hwan Ko, Myoung Jae Seo, Sung Min Kim, Hong Seok Lim

**Affiliations:** 1 Department of Regulatory Science for Medical Device, Dongguk University, Goyang 10326, Republic of Korea; jjh30512@naver.com (J.H.J.); tjfwogh990715@gmail.com (J.H.S.); rheoghks6393@naver.com (D.H.K.); tjaudwo45@naver.com (M.J.S.); 2 Department of Biomedical Engineering, Dongguk University, Goyang 10326, Republic of Korea; 3 Research Institute for Commercialization of Biomedical Convergence Technology, Dongguk University, Goyang 10326, Republic of Korea

**Keywords:** rib cage model, fatigue fractures, CPR (cardiopulmonary resuscitation), OHMCPR (out-of-hospital manual cardiopulmonary resuscitation), DOE (design of experiments), orthogonal array testing, metamodel

## Abstract

Cardiopulmonary resuscitation (CPR) is a life-saving technique used in emergencies when the heart stops beating, typically involving chest compressions and ventilation. Current adult CPR guidelines do not differentiate based on age beyond infancy and childhood. This oversight increases the risk of fatigue fractures in the elderly due to decreased bone density and changes in thoracic structure. Therefore, this study aimed to investigate the correlation and impact of factors influencing rib fatigue fractures for safer out-of-hospital manual cardiopulmonary resuscitation (OHMCPR) application. Using the finite element analysis (FEA) method, we performed fatigue analysis on rib cage models incorporating chest compression conditions and age-specific trabecular bone properties. Fatigue life analyses were conducted on three age-specific rib cage models, each differentiated by trabecular bone properties, to determine the influence of four explanatory variables (the properties of the trabecular bone (a surrogate for the age of the subject), the site of application of the compression force on the breastbone, the magnitude of applied compression force, and the rate of application of the compression force) on the fatigue life of the model. Additionally, considering the complex interaction of chest compression conditions during actual CPR, we aimed to predict rib fatigue fractures under conditions simulating real-life scenarios by analyzing the sensitivity and interrelation of chest compression conditions on the model’s fatigue life. Time constraints led to the selection of optimal analysis conditions through the use of design of experiments (DOE), specifically orthogonal array testing, followed by the construction of a deep learning-based metamodel. The predicted fatigue life values of the rib cage model, obtained from the metamodel, showed the influence of the four explanatory variables on fatigue life. These results may be used to devise safer CPR guidelines, particularly for the elderly at a high risk of acute cardiac arrest, safeguarding against potential complications like fatigue fractures.

## 1. Introduction

Acute cardiac arrest is a state in which the heart’s function to circulate blood abruptly ceases, leading to the dysfunction of body organs. In the event of such an emergency, multiple organs can suffer severe damage due to the lack of blood supply. Particularly for the brain, an interruption of oxygen supply for more than a few minutes can lead to fatal damage and aftereffects; if the golden window of opportunity is missed, it can result in brain death or even a fatality. Therefore, cardiopulmonary resuscitation (CPR) must be performed promptly and accurately on patients with acute cardiac arrest, as it can significantly increase the survival rate by two- to threefold, making it a critical emergency intervention [1,2,3]. Acute cardiac arrest occurs suddenly, and since it requires immediate emergency response by the initial discoverer, most people are required to receive CPR training, which pertains to out-of-hospital manual cardiopulmonary resuscitation (OHMCPR). However, as the primary performers of OHMCPR are typically laypeople rather than trained professionals, they are not adept at performing OHMCPR, which can also lead to side effects in the course of performing CPR, one of the most common ones being fatigue fractures due to repetitive chest compressions [4,5,6]. While the artificial external pressure from chest compressions aids in circulating blood as a substitute for the stopped heart, the ribs can fracture if the pressure required to circulate the blood is repetitively applied. In actual CPR situations, the incidence rate of rib fractures varies by age group, reported to be as low as 35% and as high as 96%, with higher rates observed in older age groups [7,8,9,10,11,12,13]. As age increases, bone density decreases, leading to a general reduction in physical strength [14,15]. Aging also results in decreased respiratory muscle capability, reduced compliance, and diminished elastic recoil, leading to an increased angle between the spine and ribs, known as the ‘barrel chest’ phenomenon. This change in thoracic shape alters the relative positions between the organs and thorax, making the ribs more susceptible to fractures from chest compressions [16]. Therefore, even when applying the same amount of force to the same chest location according to the adult CPR guidelines, the position of the organs being compressed can vary due to differences in thoracic shape by age group, and the elderly, with their decreased bone density, are at a higher risk of rib fractures. While younger patients can relatively easily recover from the side effects of CPR through rest, for elderly patients, it can lead to serious life-threatening issues. However, the adult CPR guidelines do not specifically accommodate for age-related characteristics. CPR guidelines are established or revised to suit the country in question, referencing recommendations from the International Liaison Committee on Resuscitation (ILCOR) [17,18]. For instance, the American Heart Association (AHA) encompasses a broad age range in its adult CPR guidelines, considering ‘post-puberty’ as the criterion for adulthood. Research on CPR methods considering age-specific characteristics has shown differences in the position of heart compression by age group according to the AHA guidelines. For individuals over 70, it is recommended to adjust the compression site 1 cm below the guideline-specified site, considering the position of the left ventricular outflow. This recommendation from the study suggests a method suitable for efficient blood circulation in elderly patients but does not account for secondary damage such as fatigue fractures. Therefore, for the design of efficient and safe CPR methods, consideration must also be given to major influencing factors and their impact, including rib fatigue fractures. In this study, we focus on OHMCPR, and any mention of CPR in the text refers to OHMCPR.

Existing research on rib fractures has been conducted using various methods, including cadaveric testing and finite element analysis (FEA) for fracture prediction [19,20]. However, the use of cadaveric ribs presents challenges in repeatability and there is limited availability of specimens, making it difficult to apply diverse condition changes. As an alternative, FEA has been extensively utilized. To enhance the reliability of FEA results, several studies have validated rib cage FE models by comparing them with actual experimental outcomes. For instance, Iraues et al. developed a rib FE model to analyze the risk of rib fractures during impacts and validated their model through comparison with real-world experimental data [21]. Additionally, Vavalle et al. utilized a human body FEA model to simulate impacts to the chest and abdomen under various localized conditions, verifying the accuracy of their model by demonstrating its consistency with experimental data [22]. Traditionally, most research on rib fractures has focused on those resulting from collisions or other accidents [23,24,25,26,27,28,29]. Unlike these studies, rib fractures occurring during cardiopulmonary resuscitation (CPR) represent a unique situation where loads are repetitively applied. The purpose of this study was to determine the potential fatigue fractures that may occur during CPR by designing a finite element model encompassing the entire thoracic structure composed of numerous bones. Using FEA, we conducted fatigue analysis on thoracic structure models, incorporating different trabecular bone properties across various age groups and applying varying conditions of chest compression intensity, location, and frequency. To mimic the complex interaction of chest compression conditions present in actual CPR scenarios, we examined the fatigue life sensitivity and the interaction between load condition parameters based on diverse combinations. Considering the extensive time and costs associated with finite element analysis across all potential cases, we employed orthogonal array testing from the design of experiments (DOE) to minimize experimental size while selecting only meaningful cases for analysis. The results of this analysis formed the basis for developing a deep learning-based metamodel to estimate fatigue life values across all cases, further analyzing the sensitivity and the influence of load condition parameters across different age groups. The findings revealed significant differences in sensitivity and interaction among parameters by age group, suggesting the possibility of developing safer CPR methods for the elderly, who are at a higher risk of acute cardiac arrest, while considering the risk of fatigue fractures.

## 2. Methods

### 2.1. Finite Element Model Development

**Figure 1:**
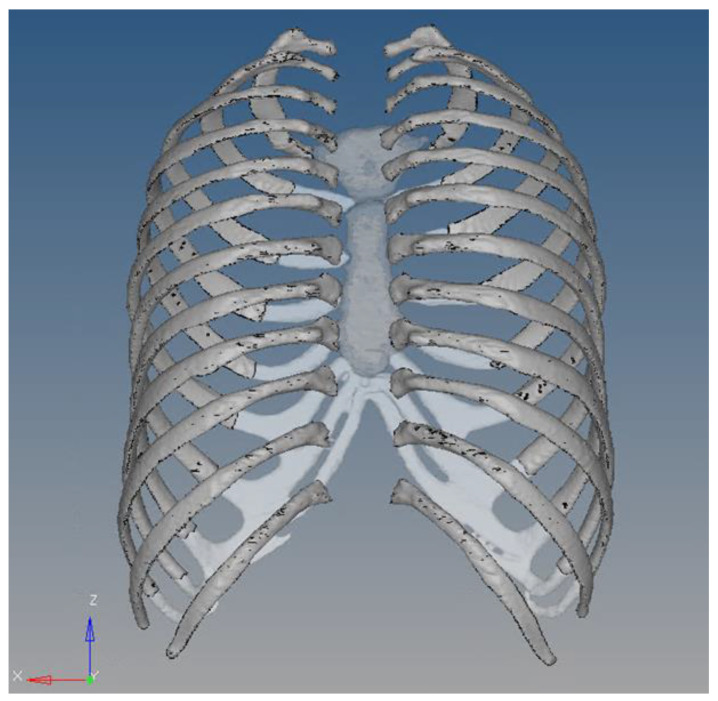
Geometry of the rib cage with the spinal section removed.

**Figure 2:**
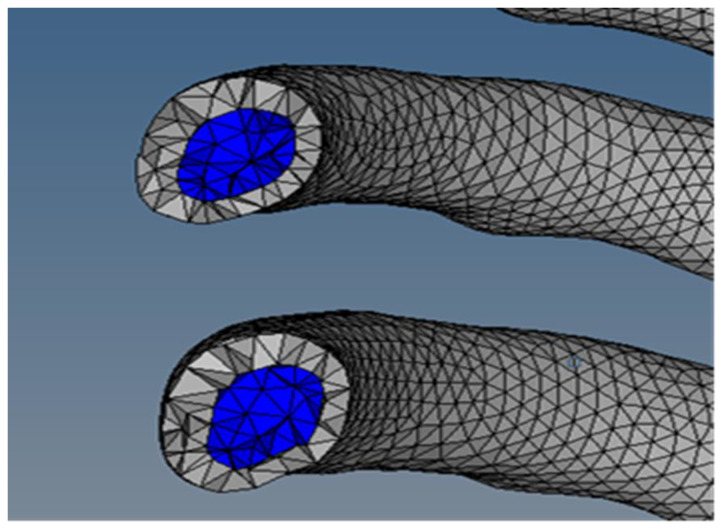
The gray area represents the cortical bone, and the blue area represents the trabecular bone.

The rib cage model was designed using whole-body CT data from a male in his 40s who was a healthy person with no musculoskeletal problems. The 3D thoracic model was provided by Materialise Inc. (Leuven, Belgium), and ribs, cartilage, and spine were separated from this model to create the rib cage model. The geometrical information for this 3D model provided by Materialise Inc. is detailed in Table 1. In the development of the finite element rib cage model used in this study, the spine was excluded, as negligible impact on rib fatigue life was anticipated. This is illustrated in Figure 1, showing the rib cage geometry sans the spinal section. Subsequently, as shown in Figure 2, the boundary between the trabecular and cortical bones was delineated. To address the challenge of irregular model shapes caused by the inherent resolution limitations of CT data. We utilized 3-matic software, version 18.0 (Materialise Inc., Leuven, Belgium), applying its wrap function. This function helps smooth out the model’s surface to address incomplete model shapes. To accurately set the cortical bone thickness, a previous study that utilized CT data to measure the thickness of each bone layer was consulted, establishing the cortical bone’s thickness at approximately 2.5 mm [30].

The preprocessing for the finite element analysis was carried out using the Hyperworks software, version 2023 (Altair Engineering Inc., Troy, MI, USA). The mesh size was determined to be 2.0 mm, taking into account both the accuracy of the analysis and computational efficiency [31,32,33]. The finite element analysis and post-processing stages employed the use of Optistruct software, version 2023 (Altair Engineering Inc., Troy, MI, USA).

**Figure 3:**
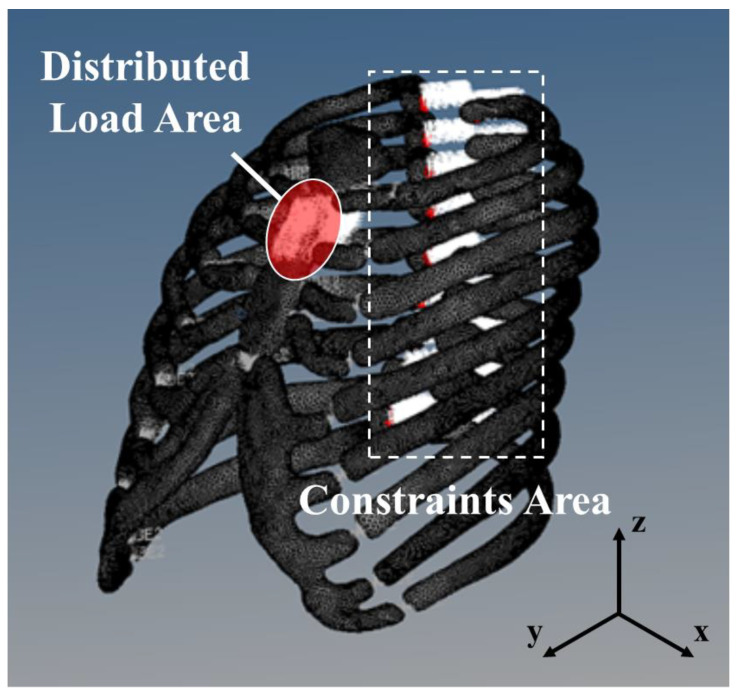
Distributed load area and constraints area.

The designated areas for applying load and constraints in the finite element analysis are shown in Figure 3. To simulate the load applied during cardiopulmonary resuscitation (CPR), the sagittal axis was aligned with the *y*-axis, and a vertically distributed load was applied along this axis. In accordance with the American Heart Association (AHA) CPR guidelines, the load was centered at the midpoint of the sternum [34,35]. Given that chest compressions in out-of-hospital CPR are performed using the palm, the load was distributed in a manner reflective of the palm size. Constraints were designed to mimic a patient lying flat on his/her back and to account for the thoracic mobility induced by CPR forces; restrictions were applied to rotational degrees of freedom along the *x*, *y*, and *z* axes. Furthermore, connections between bones and between bones and rib cartilage were modeled as rigid elements to accurately replicate the structural integrity and mechanical behavior of the thoracic region.

### 2.2. Fatigue Fracture Analysis

To conduct fatigue analysis using finite element analysis software, the Hyperworks software, version 2023 (Altair Engineering Inc., Troy, MI, USA), it is crucial to define analysis parameters that consider the material and fatigue characteristics. Taking into account the fatigue load conditions occurring during cardiopulmonary resuscitation (CPR) and the properties of the ribs, relevant analysis parameters were established. Unlike typical fatigue loads, the fatigue load during CPR is characterized by repetitive compressive forces of a constant magnitude at a fixed frequency, ranging up to 10,000 cycles, indicative of low-cycle fatigue (LCF) conditions. To suit these characteristics, the Strain-Life Approach (E-N Approach) was employed, which is appropriate for conditions where plastic deformation occurs due to low-cycle fatigue and repetitive loading [36].

Given that ribs exhibit brittle material properties, easily fracturing even under minor deformations, the Maximum Principal Stress Theory was utilized to calculate the yield stress [37]. Moreover, as bones are more susceptible to failure under tensile stress than compressive stress, indicating a physical property where fractures occur more readily under tension, Smith, Watson, and Topper’s Damage Parameter was applied to calculate fatigue life caused by tensile stress [38,39,40]. For the mean stress correction method, the Goodman Theory was adopted, which is suitable for brittle materials like bone [39,40].

### 2.3. Finite Element Analysis Conditions

In the present study, the parameters that could influence rib fatigue fractures during CPR were taken to be age-related bone density, chest compression magnitude, chest compression site, and chest compression rate. Different values of these parameters were used (Table 2). Condition 1 focuses on analyzing rib fatigue fractures across different age groups by applying adult CPR guidelines, highlighting bone density variations. Different trabecular bone material properties were applied to rib cage models for three age groups, Young, Middle, and Old, and fatigue analysis was conducted based on AHA adult CPR guideline conditions. Conditions 2 to 4 investigate the effects of variations in chest compression conditions that influence the fatigue life of ribs, analyzing how changes in load magnitude, load site, and load rate affect the fatigue life of the rib cage model, considering age-specific bone properties. Load condition parameters were set based on the values derived from AHA adult CPR guidelines. Detailed descriptions for Conditions 1 to 4 are provided in the following sections.

#### 2.3.1. Material Properties of Bone Age

The decrease in bone density with age leads to a weakening of the bone’s physical strength and increased vulnerability to external loads [42]. This study sought to analyze the differences in rib fatigue fracture patterns across different age groups when applying identical load intensity to the same location on the chest, following adult CPR guidelines. To this end, based on variations in trabecular bone properties, the analysis was categorized into three groups: Young (ages 16–39), Middle (ages 40–59), and Old (ages 60–83). Despite a decrease in both cortical and trabecular bone densities with age, the density differences are predominantly attributed to trabecular bone variations; thus, constant material properties were applied to the cortical bone and rib cartilage across all groups [31,41]. Accordingly, the material properties applied to the rib cartilage and cortical bone in the age-specific rib cage model are as listed in Table 3 [41], while the material properties for the trabecular bone, varied by age group, are detailed in Table 4 [43]. The conditions for load magnitude, load site, and load rate were established based on the American Heart Association (AHA) guidelines at 500 N, the center of the sternum, and 110 compressions per minute, respectively [34,35].

#### 2.3.2. Magnitude of Compression Force

According to the guidelines of the American Heart Association (AHA), it is recommended to compress the chest of a person undergoing cardiac arrest to a depth of about 5 cm during CPR. Furthermore, to avoid complications, compressions should not exceed a depth of 6 cm. In this study, a static analysis of the rib cage finite element analysis (FEA) model was performed to estimate the force required to achieve a deformation depth of 5 cm. The static analysis determined that the load required for the recommended compression depth of 5 cm was approximately 500 N, while the force corresponding to a depth of 6 cm was about 550 N. Based on these findings, the load magnitudes were set to 450 N, 500 N, and 550 N, respectively. Other load conditions, such as load site and load rate, were applied following the guideline standards, specifically at the recommended compression location (the center of the sternum) and 110 compressions per minute, respectively.

#### 2.3.3. Compression Site

**Figure 4:**
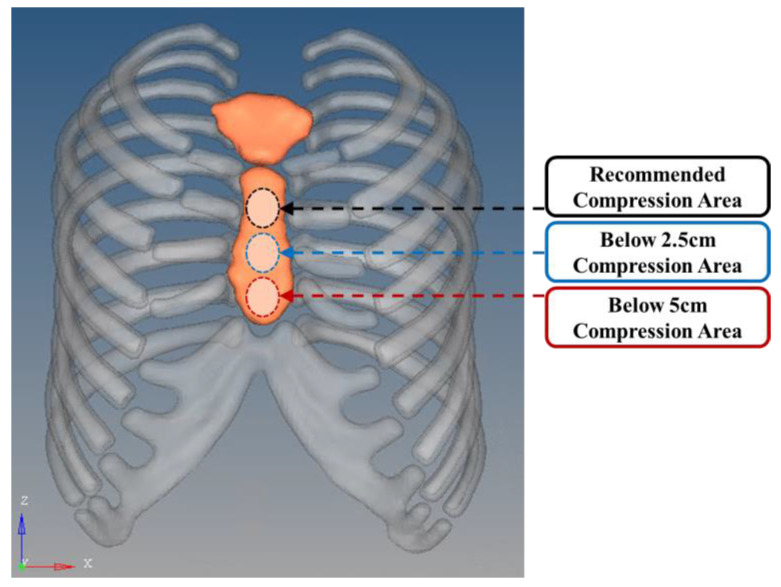
Compression site of guideline area (black), below 2.5 cm area (blue), and below 5 cm area (red).

The load site was selected based on the recommended compression site at the center of the sternum, as specified in the American Heart Association (AHA) guidelines. Considering that CPR is an emergency procedure often performed by untrained bystanders in urgent situations, additional intervals of −2.5 cm and −5 cm from the recommended compression site were established to account for potential deviations in compression placement. Figure 4 illustrates the recommended compression area as per AHA guidelines on the rib cage model, as well as areas 5 cm below the recommended site. The other load conditions, namely, load magnitude and load rate, were applied according to the guideline standards, set at 500 N and 110 compressions per minute, respectively.

#### 2.3.4. Compression Rate

Based on the American Heart Association (AHA) adult CPR recommendation of 100 to 120 compressions per minute, an average rate of 110 compressions per minute was set as the load rate for this study. To further explore the fatigue life under varying conditions, additional load frequencies of 80 and 140 compressions per minute were also examined, diverging from the guideline’s recommended range. The other load conditions, such as load magnitude and load site, followed the guideline’s recommendation, applying a load of 500 N and a load site at the center of the sternum.

### 2.4. Comprehensive Influence Analysis

#### 2.4.1. Sensitivity of Load Condition Parameters and Correlations across Age Groups

To investigate the impact of variations in bone properties and load condition parameters (magnitude, site, and rate) on fatigue life across different age groups, each load condition was individually altered while applying guideline-recommended values for the remaining conditions. However, in actual CPR situations, it is not possible to independently control each load condition. Therefore, to analyze various possible scenarios during CPR and enhance the similarity with real-life situations, load conditions were cross-examined under combined load circumstances. This approach aimed to investigate the age-specific fatigue life outcomes and to analyze the sensitivity of fatigue life to load condition parameters and the interrelation between these parameters across different age groups. The analysis was conducted using PIAnO 2024 software (PIDOTECH Inc., Seoul, Republic of Korea).

Considering that chest compression depth and location are relatively easy for the practitioner to adjust according to guidelines during CPR, the variability of the chest compression rate, which lacks a method for self-verification by the practitioner and can be subject to biases in emergency situations, was deemed to potentially have a broader range in actual CPR. The compression rate, recommended to be between 100 to 120 compressions per minute according to guidelines, was explored within this range (100, 110, and 120 compressions per minute) and extended beyond to include rates considered outside the recommended range (80 and 140 compressions per minute).

#### 2.4.2. Metamodel Construction for Fatigue Life Prediction across All Conditions

The defined analysis cases for the age-specific combined load conditions are listed in Appendix A, comprising a total of 135 scenarios.

In this study, orthogonal array testing, a design of experiments method, was applied to analyze fatigue life under various complex load conditions across age groups. Due to the absence of an appropriate orthogonal array for each of the 45 cases per age group, a total of 135 cases were required to cover all analysis scenarios. However, conducting finite element analysis for all cases was deemed time-consuming and costly. To circumvent this, a metamodel was constructed using selected data to predict fatigue life. The selection process, facilitated by PIAnO 2024 software (PIDOTECH Inc., Seoul, Republic of Korea), yielded 45 analysis cases that satisfied orthogonality across all scenarios [44]. These selected cases, detailed in Appendix B, were then subjected to fatigue analysis.

Based on the finite element analysis results of the selected 45 cases, a deep learning-based metamodel was constructed using PIAnO 2024 Metamodeler software (PIDOTECH Inc., Seoul, Republic of Korea). This metamodel enabled the prediction of fatigue life for all 135 cases, aiming to analyze the sensitivity of fatigue life to load condition parameters and interrelation between load condition parameters across different ages. This approach was predicated on the assumption that performing CPR in areas of lower sensitivity to load condition parameters could potentially be safer from secondary injuries such as fatigue fractures. The PIAnO 2024 Metamodeler software, by analyzing data types and forms, recommends the optimal metamodel, automatically setting the hyperparameters and architecture. In the case of this study, among various models, deep learning models were given priority. The hyperparameters for the optimized deep learning model, as determined after a thorough analysis, are outlined in Table 5. Following the assessment of reliability based on the predicted values from the deep learning-based metamodel trained on 45 analysis cases, this metamodel was used to investigate the sensitivity of fatigue life to load condition parameters across different ages and the interrelation between load condition parameters on age-specific fatigue life, based on the fatigue life predictions for all 135 cases.

## 3. Results

During cardiopulmonary resuscitation (CPR), the rib cage model was subjected to fatigue analysis using age-specific bone properties and three load conditions (load site, magnitude, and rate) as parameters, simulating adult CPR as per the American Heart Association (AHA) guidelines. To assess fatigue life under standard adult CPR conditions, the load magnitude, site, and rate were set to 500 N, midline at the lower half of the sternum, and 110 compressions/min, respectively. Under these load conditions, the Young, Middle, and Old groups exhibited fatigue life values of 3172, 1729, and 788 cycles, respectively. Compared to the fatigue life of 3172 cycles for the Young group, the Middle group showed a reduction of about 54%, and the Old group showed a reduction of about 45% compared to the Middle group.

### 3.1. Fatigue Life

**Figure 5:**
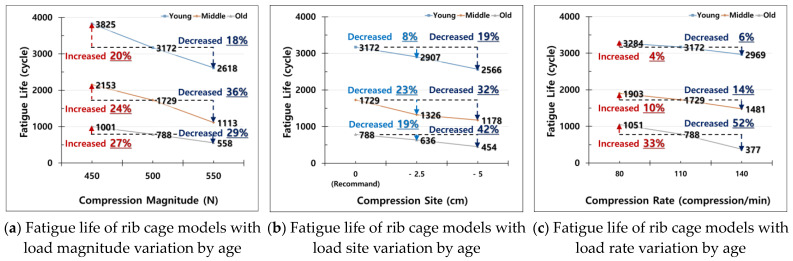
Fatigue life of rib cage models with load conditions variation by age. (**a**–**c**) all apply the following load conditions, except for the respective load magnitude, load site, and load rate values: the load magnitude is 500 N, which corresponds to the recommended chest compression depth from the AHA adult CPR guidelines; the load site is the midpoint of the sternum’s lower half; and the load rate is 110 compressions per minute, which is the average value of the recommended chest compression rate of 100–120 compressions per minute.

The fatigue life values of rib cage models with age-specific bone properties were investigated for the three load conditions (load magnitude, site, and rate) set as parameters (Figure 5). The fatigue analysis results, based on changes in load magnitude, revealed that fatigue life increased at a load magnitude of 450 N and decreased at 550 N, compared to the recommended 500 N load for adult CPR guidelines, across all groups. Fatigue life for each age group, relative to the 500 N recommended condition, increased by factors of 1.20, 1.24, and 1.27 at 450 N for the Young, Middle, and Old groups, respectively, and decreased by factors of 0.82, 0.64, and 0.71 at 550 N (Figure 5a). The increase in fatigue life for a 450 N load magnitude was similar across all age groups, but the reduction in fatigue life at 550 N differed by age group. The Middle group exhibited the greatest decrease in both the numerical value and percentage of fatigue life with a 550 N load magnitude.

Second, the fatigue life values of rib cage models with age-specific bone material properties were evaluated by varying the load site, using the recommended chest compression point in the AHA adult CPR guidelines as a reference (Figure 5b). Fatigue life decreased as the load application point moved away from the center of the sternum, but the extent of reduction varied by age group and was not linearly proportional to changes in load site. Compared to the fatigue life at the recommended load site, the fatigue life decreased by factors of 0.92, 0.77, and 0.81 at −2.5 cm, and by 0.81, 0.68, and 0.58 at −5 cm, for the Young, Middle, and Old groups, respectively. The greater decrease percentage in fatigue life for the Middle and Old groups compared to the Young group indicates that the load site parameter has a larger impact on fatigue life in these groups. The Middle group experienced a sharp reduction in fatigue life at −2.5 cm, likely due to different sensitivities to parameter changes in different regions.

Lastly, the fatigue life values of rib cage models with age-specific bone properties were assessed by varying the load rate, using the average recommended compression rate of 110 compressions/min from the AHA adult CPR guidelines (Figure 5c). Compared to the fatigue life at this load rate, the fatigue life increased by factors of 1.04, 1.10, and 1.33 at 80 compressions/min, and decreased by 0.94, 0.86, and 0.48 at 140 compressions/min, for the Young, Middle, and Old groups, respectively. The largest variation in fatigue life occurred in the Old group, which showed a 52% decrease at 140 compressions/min, indicating that this group was the most sensitive to changes in load rate.

### 3.2. Sensitivity of Fatigue Life

In independently controlled analysis conditions of load condition parameter values, the fatigue life of the rib cage model with age-specific bone properties was assessed. However, in real CPR scenarios, chest compression conditions cannot be controlled independently. To simulate more realistic scenarios, various load condition parameters were cross-analyzed. Out of a total of 135 analysis cases, 45 were chosen via the orthogonal array method, and a deep learning-based metamodel was used to predict fatigue life values for all 135 cases. With an R2 value of 0.999999977, the metamodel was deemed reliable. Based on predicted fatigue life values, its sensitivity to load condition parameters and their correlations were analyzed by age group.

**Figure 6:**
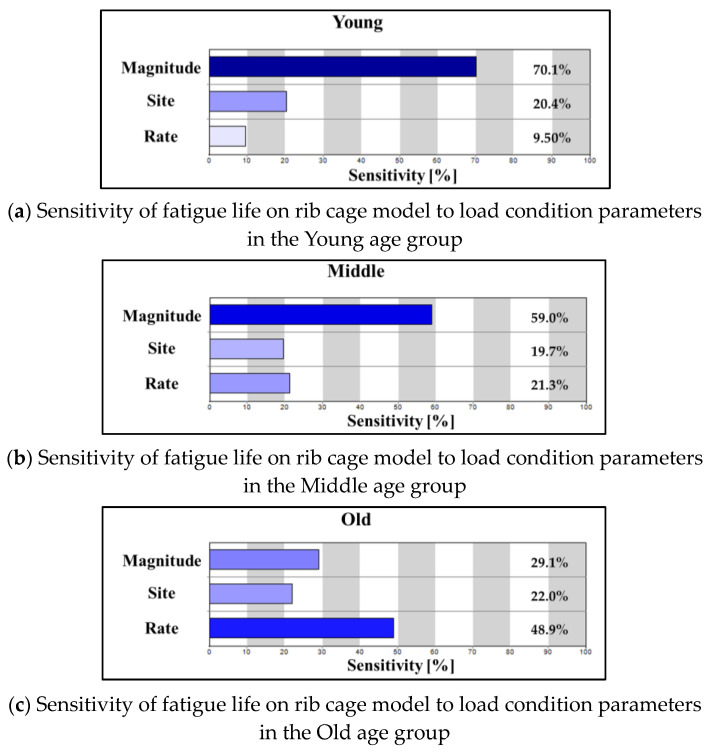
Sensitivity analysis of load condition parameters on rib cage model fatigue life by age.

Figure 6 shows the sensitivity of fatigue life to each load condition parameter when the influence of the three load conditions is normalized to 100%. In the Young group, fatigue life sensitivity to load magnitude, load site, and load rate was 70.1%, 20.4%, and 9.50%, respectively, showing the highest sensitivity to load magnitude (Figure 6a). In the Middle group, fatigue life sensitivity was 59.0%, 19.7%, and 21.3%, respectively, indicating that sensitivity to load magnitude is still the highest, similar to the Young group, although it is relatively lower at 59.0%. A notable difference is that fatigue life sensitivity to load rate is higher than sensitivity to load site. Finally, in the Old group, unlike the previous two groups where sensitivity to load magnitude was the highest, sensitivity to load rate was 48.9%, showing that load rate has the greatest influence on fatigue life (Figure 6c).

By analyzing fatigue life for the rib cage model with age-specific bone properties, independently controlling the values of load condition parameters (Figure 5), and investigating the sensitivity of fatigue life to load condition parameters (Figure 6), we observed trends but not complete numerical consistency. We analyzed the correlations between load condition parameters to understand how their combined effects cause non-linear changes in fatigue life with parameter variations (Figure 5).

**Figure 7:**
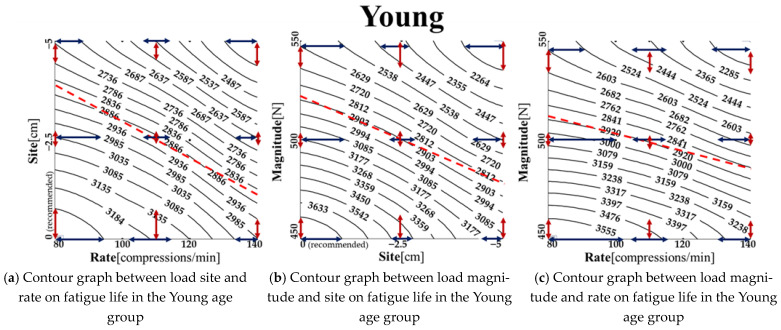

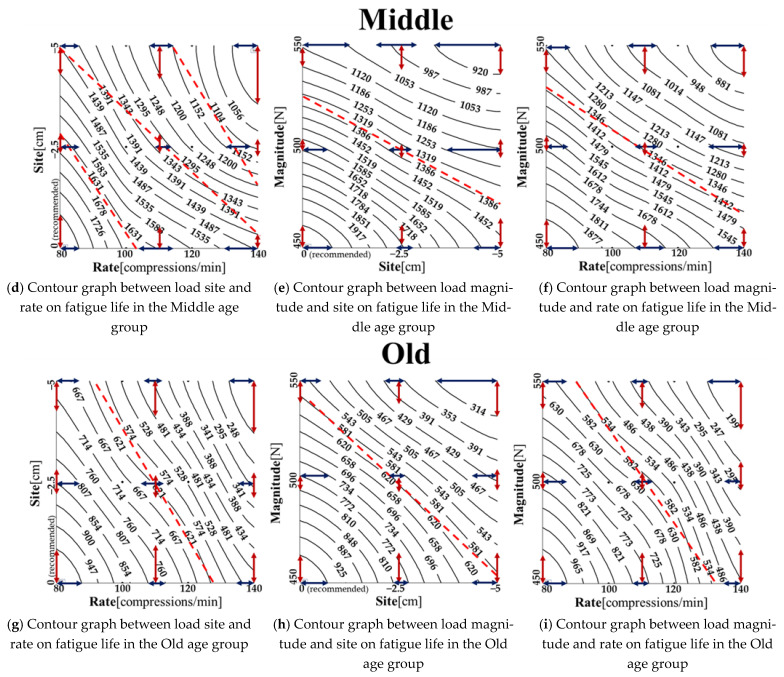
Fatigue life contour graphs illustrating the correlation between load condition parameters in the three age groups (Young, Middle, and Old).

To identify the correlation between two different load condition parameters, the remaining load condition was fixed at the recommended setting from the AHA adult CPR guidelines. We then analyzed the fatigue life values determined by changes in the other two load conditions. Figure 7 shows the contour graph depicting fatigue life values influenced by these two load condition parameters. The black lines connect points with identical fatigue life values, while the red dashed line indicates the trend line on the contour graph. The arrows represent the distance between contour lines, with shorter arrows implying greater sensitivity in fatigue life to changes in load condition parameters. The trend line reveals the overall inclination of the contour graph, leaning towards the axis of the load condition parameter that has a greater influence on fatigue life changes.

Figure 7a–c are contour graphs that illustrate the correlation of different load condition parameters in the young age group. Figure 7a shows the correlation between load site and load rate. The trendline on the contour graph leans towards the load site, consistent with Figure 6a, indicating a greater sensitivity to changes in load site. Near the recommended site (0 cm), as load rate increases, sensitivity to both load site and load rate grows. At −2.5 cm from the recommended site, sensitivity to load site increases significantly, but the sensitivity to load rate remains relatively unchanged despite increases in load rate. At approximately −5 cm, sensitivity to load site decreases, but is still higher than at 0 cm, and sensitivity to load rate remains fairly constant regardless of load rate increases. Figure 7b displays the correlation between load magnitude and load site in the Young age group. Sensitivity to load magnitude is highest at 70.1%, as evidenced by the trend line sloping towards the load magnitude axis, reflecting Figure 6a. When load magnitude remains constant, the sensitivity to load site increases and then decreases as load site moves downward from the recommended 0 cm site, while sensitivity to load magnitude remains fairly constant. With constant load site, sensitivity to load magnitude increases and then decreases with rising load magnitude.

Figure 7c illustrates the correlation between load magnitude and load rate in the Young age group. The trend line slopes towards the load magnitude axis, indicating a greater overall impact of load magnitude on fatigue life than load rate. When load magnitude is less than 500 N (translated from the recommended chest compression depth), sensitivity to load rate increases before stabilizing with rising load rate, while sensitivity to load magnitude remains steady. Around 550 N, sensitivity to both load magnitude and load rate remains consistent despite increasing load rate.

Figure 7d–f present the contour graphs that illustrate the correlation between different load condition parameters for the Middle age group. Figure 7d focuses on the correlation between load site and load rate. The sensitivity difference between load site and load rate is only 1.7%, so the trendline slope is close to 1 (Figure 6b). When comparing different load rates to the recommended rate of 110 compression/min, the trendlines for high and low rates diverge, tilting towards the load rate axis. This shows that fatigue life sensitivity to load rate is more pronounced at these levels. As the load site moves downward from 0 cm (recommended site) at a rate of 80 compressions/min, sensitivity to the load site initially increases but then decreases, while sensitivity to load rate remains fairly stable. Near the recommended rate of 110 compressions/min, sensitivity to both load site and load rate increases and then decreases. At 140 compressions/min, sensitivity to load rate remains stable while sensitivity to load site decreases.

Figure 7e shows the contour graph indicating the correlation between load magnitude and load site. The trendline tilts towards the load magnitude axis due to its highest sensitivity (Figure 6b), implying that fatigue life is more sensitive to changes in magnitude. When load magnitude is constant, moving the load site below 0 cm (recommended site) reduces sensitivity to load magnitude, while sensitivity to load site first increases and then decreases. When the load site is kept constant, sensitivity to load magnitude increases and then decreases as magnitude increases.

Figure 7f illustrates the correlation between load magnitude and load rate in the middle age group. Similar to Figure 7e, the trendline tilts towards the load magnitude axis due to higher sensitivity to magnitude. When the load magnitude is around 450 N, increasing load rate results in a slight increase in sensitivity to magnitude, while sensitivity to load rate remains constant. At a load magnitude of 550 N, sensitivity to load magnitude shows a slight increase. In this region, increasing the load rate leads to a slight decrease in sensitivity to both load site and rate, but both remain largely constant. Similar to Figure 7e, sensitivity to load magnitude increases and then decreases as magnitude increases when the load site is kept constant.

Figure 7g–i show the contour graphs illustrating the correlation between different load condition parameters for the Old age group. Unlike other age groups, the Old age group shows the highest fatigue life sensitivity to load rate (Figure 6c), which influences the shape of its contour graph. Figure 7g depicts the correlation between load site and load rate. As indicated in Figure 6c, with fatigue life sensitivity to load rate at 48.9%, the trendline tilts towards the load rate axis, indicating that fatigue life changes are most sensitive to load rate variations. When load rate remains constant, as the load site moves down from 0 cm (the recommended site), sensitivity to load rate remains high and fairly stable, while sensitivity to load site initially increases and then decreases.

Figure 7h depicts the contour graph showing the correlation between load magnitude and load site for the Old age group. These two load condition parameters have the smallest difference in sensitivity in the Old group (Figure 6c), which makes the overall trendline almost level, indicating that fatigue life is equally influenced by both parameters. In regions where load magnitude and load site have constant values, sensitivity to the other load condition parameter first increases and then decreases.

Figure 7i shows the correlation between load magnitude and load rate for the Old age group. Similar to Figure 7g, the trendline tilts towards the load rate axis due to the highest fatigue life sensitivity. When load rate remains constant, increasing load magnitude leads to a decrease in sensitivity to load rate around 140 compressions/min, while sensitivity to load magnitude first increases and then decreases.

## 4. Discussion

In this study, we developed a model capable of predicting rib fatigue fractures across different age groups during CPR situations, applying age-specific bone property data. To simulate real-life emergency scenarios, the fatigue life values of the rib cage model under various load conditions were examined, and based on the resultant data, we identified the load conditions that predominantly affect the fatigue life across different age groups, namely, the sensitivity of fatigue life parameters. Additionally, through analyzing the fatigue life variation trend lines against two other load condition parameters, we were able to determine the interrelations between load condition parameters.

To examine the patterns of fatigue fractures according to age-specific bone density, the analysis under condition 1, applying load conditions specified in the American Heart Association (AHA) CPR guidelines, was segmented into three groups based on changes in trabecular bone properties: Young, Middle, and Old. The analysis revealed fatigue fractures occurring at 3172, 1729, and 788 repetitions of load application, respectively. Given that acute cardiac arrest significantly impacts age and is prevalently observed in the elderly, they can be considered a primary target group for adult CPR. Although individual variations exist, the fatigue life in the Old group showed a significant decrease to about 0.25 times that of the Young group, indicating the need for further research to develop age-specific guidelines considering the safety from fatigue fractures.

In the analysis of conditions 2 to 4, applying varied load magnitude, load site, and load rate, the maximum fatigue life appeared at the recommended load site (center of the sternum), the minimum load magnitude, and the minimum load rate. By crossing various load condition parameters to simulate situations akin to actual CPR in the analysis condition of combined load conditions, all groups uniformly showed the highest fatigue life under conditions of minimum load magnitude, recommended load site (center of the sternum), and minimum load rate. While the condition inducing the least fatigue life for the maximum fatigue life is predictably result-oriented, the analysis of fatigue life parameter sensitivity and the interrelation between load condition parameters based on the fatigue life data from each analysis condition yielded significant findings. Though the optimal load conditions for maximum fatigue life were the same across all groups, differences existed in the dominant load conditions (load magnitude, load site, and load rate) affecting the fatigue life and their impact levels. In the Young and Middle groups, the rib cage model’s fatigue life showed the highest sensitivity to changes in load magnitude, with the sensitivity level in the Young group being approximately 11.1% higher than in the Middle group. Contrarily, the Old group displayed the highest sensitivity to the load rate condition, suggesting that for elderly subjects undergoing CPR, reducing compression rate might be more meaningful than decreasing compression magnitude to avoid fatigue fractures.

To prevent rib fractures effectively, it is not only the most sensitive load condition parameters that should be considered but also the interplay among them. The three load conditions do not independently influence fatigue life; the impact of one load condition’s variation on fatigue life depends on the settings of the other load conditions. According to the analysis of the correlation between load condition parameters, the sensitivity of fatigue life to each load condition parameter was not consistent across all points. In particular, in the contour graph illustrating the correlation between load site and load rate in the Middle group, the trend lines for regions of high and low load rate, based on the recommended chest compression rate of 110 compressions/min, differ significantly from the overall trend line of the contour graph. This highlights how the sensitivity of fatigue life to load condition parameters can vary significantly depending on the combination of load conditions. This implies that there are points where the impact of specific load condition parameters is minimal. Therefore, in CPR studies focused on preventing fatigue fractures, it is essential to consider the interactions between different load conditions, rather than solely considering load conditions in the order of parameter sensitivity, to derive the optimal method. For instance, in all three age groups, the sensitivity of fatigue life to load site and magnitude generally increased and then decreased with changes in the values of load condition parameters. To clinically apply these trends when developing age-specific CPR guidelines, it is suggested that chest compression conditions should be determined within the range where sensitivity to the respective load condition parameter reaches its peak.

This study has several limitations. Firstly, it relies solely on a 3D model constructed from a CT scan of a single male in his forties, which restricts the generalizability of the results. Age-related changes in thoracic structure, such as the barrel chest phenomenon, can significantly affect rib fatigue life and should undoubtedly be considered in the fatigue analysis of the ribs. Additionally, considering that the average age for rib fractures resulting from out-of-hospital cardiac arrest CPR is 66 years, using a model of only a middle-aged man may not accurately reflect real-world conditions. Obtaining imaging data to verify changes in thoracic structure due to aging is challenging, and variations such as rib angle alterations are influenced by a wide range of genetic and environmental factors, making generalization difficult. The primary aim of this research was to analyze the impact of age-related changes in bone material properties on the fatigue life of the rib cage under various chest compression parameters. Therefore, no statistical generalization was performed. Fractures from manual cardiopulmonary resuscitation out of hospital typically occur in older populations; however, the study utilized a model that reflected changes in bone properties across different ages, opting for a model representing a middle age range. This choice was made because focusing solely on older age groups could significantly differ from younger thoracic structures, complicating the analysis based solely on changes in bone properties. Therefore, to design safer age-specific CPR methods that prevent rib fractures, it is necessary to develop a rib cage structural model that incorporates changes in thoracic morphology across different ages, warranting further research.

Second, it is essential to demonstrate that conditions identified as significant for preventing rib fractures do not impede the primary objective of CPR, which is to artificially circulate blood when the heart stops. Rib fractures are a secondary issue that may arise during CPR; thus, proposed methods for preventing them should not result in a decreased efficiency of cardiac resuscitation. This research did not evaluate the changes in blood circulation efficiency under load conditions optimized for maximum fatigue life. Subsequent studies must verify that methods proposed for safer CPR can achieve the same level of resuscitation efficiency as when CPR is performed according to existing guidelines.

Lastly, when setting the load site parameter, only vertical displacement was considered with reference to the adult CPR guidelines. In reality, the chest compression location may shift laterally during CPR, but in our fatigue analysis, we only varied the location along the midline of the breastbone, which may differ from actual scenarios. However, in this study, we attempted to address this limitation by applying the load in the form of a distributed load, considering the contact area of the palm during chest compression.

## 5. Conclusions

In this study, rib cage models were designed by applying age-specific bone material properties, dividing the subjects into Young, Middle, and Old groups, and analyzing the fatigue life under various load conditions. It was found that fatigue life markedly decreased in the older age groups when compared across ages. While the tendency for higher fatigue life under conditions that induce less fatigue life was consistent across all age groups, there was a notable age-specific difference in the sensitivity of fatigue life to each load condition parameter and the interplay among load condition parameters. In the Young and Middle groups, fatigue life showed high sensitivity to the magnitude of chest compression and was most influenced by this parameter. Conversely, in the Old group, fatigue life was most sensitive to the rate of chest compressions and was significantly influenced by the interplay of load condition parameters.

From the perspective of preventing rib fractures induced by CPR, it is crucial to consider the effects among different load conditions. However, it appears that for the younger and middle-aged groups, the magnitude of chest compressions should be a focal point, while for the older age group, attention should be given to the rate of chest compressions when performing CPR. Future research incorporating clinical considerations of age-related changes in thoracic structure and the efficiency of blood circulation is warranted to propose effective and safe age-specific CPR methods.

## Figures and Tables

**Table 1:** Geometrical details of the rib cage model.

Geometrical Details of the Rib Cage Model	
Rib cage width	324 mm
Rib angle	39.59°
Haller index	2.534
Sagittal cross-sectional area	644.7 cm^2^

**Table 2:** Parameters for finite element analysis, which details the specific conditions used in each model, explicitly noting variations in age-related bone material properties, compression magnitude, site, and rate, to ascertain the singular effect of each parameter [34,35,41].

		Age	Load Magnitude	Load Site	Load Rate [Compressions/min]
1. Bone material properties		Young	500 N	Recommended site	110
		Middle			
		Old			
Chest Compression Conditions	2. Magnitude of compression force	Young	450 N	Recommended site	110
		Middle	500 N		
		Old	550 N		
	3. Compression site	Young	500 N	Recommended site	110
		Middle		Below 2.5 cm	
		Old		Below 5 cm	
	4. Compression rate	Young	500 N	Recommended site	80
					100
		Middle			110
					120
		Old			140

In each condition, a gray background color for the variables indicates the independently controlled variable.

**Table 3:** Mechanical properties of the rib cartilage and cortical bone [41].

	Rib Cartilage	Cortical Bone
Compressive modulus [MPa]	0.45~0.80	24,000

**Table 4:** Age-related variation in the mechanical properties of the trabecular bone [43].

	Young	Middle	Old
Compressive modulus [MPa]	654	829	613
Compressive strength [MPa]	10.6	9.86	7.27
Ultimate compressive strain [%]	2.48	2.12	2.05

**Table 5:** The hyperparameters for the optimized deep learning model.

Hyperparameter	Setting
Activation Function	ReLU
Optimization Method	Stochastic Gradient Descent
Tolerance for Optimization	0.0001
Momentum	0.9
Batch Size	Default
Learning Rate	0.01
Maximum Number of Iterations	200

## Data Availability

The raw data supporting the conclusion of this article will be made available by the authors on request.

## Appendix A

**Table A1:** All 135 analysis cases for age-specific combined load conditions.

Age	Load Magnitude	Load Site	Load Rate [Compressions/min]
Young	450 N	Recommended site	80, 100, 110, 120, 140
		Below 2.5 cm	80, 100, 110, 120, 140
		Below 5 cm	80, 100, 110, 120, 140
	500 N	Recommended site	80, 100, 110, 120, 140
		Below 2.5 cm	80, 100, 110, 120, 140
		Below 5 cm	80, 100, 110, 120, 140
	550 N	Recommended site	80, 100, 110, 120, 140
		Below 2.5 cm	80, 100, 110, 120, 140
		Below 5 cm	80, 100, 110, 120, 140
Middle	450 N	Recommended site	80, 100, 110, 120, 140
		Below 2.5 cm	80, 100, 110, 120, 140
		Below 5 cm	80, 100, 110, 120, 140
	500 N	Recommended site	80, 100, 110, 120, 140
		Below 2.5 cm	80, 100, 110, 120, 140
		Below 5 cm	80, 100, 110, 120, 140
	550 N	Recommended site	80, 100, 110, 120, 140
		Below 2.5 cm	80, 100, 110, 120, 140
		Below 5 cm	80, 100, 110, 120, 140
Old	450 N	Recommended site	80, 100, 110, 120, 140
		Below 2.5 cm	80, 100, 110, 120, 140
		Below 5 cm	80, 100, 110, 120, 140
	500 N	Recommended site	80, 100, 110, 120, 140
		Below 2.5 cm	80, 100, 110, 120, 140
		Below 5 cm	80, 100, 110, 120, 140
	550 N	Recommended site	80, 100, 110, 120, 140
		Below 2.5 cm	80, 100, 110, 120, 140
		Below 5 cm	80, 100, 110, 120, 140

## Appendix B

**Table A2:** Orthogonal array of 45 analysis cases.

Age	Load Magnitude	Load Site	Load Rate [Compressions/min]
Young	450 N	Recommended site	80, 100
		Below 2.5 cm	110, 120
		Below 5 cm	140
	500 N	Recommended site	80, 100
		Below 2.5 cm	110
		Below 5 cm	120, 140
	550 N	Recommended site	80
		Below 2.5 cm	100, 110
		Below 5 cm	120, 140
Middle	450 N	Recommended site	120, 140
		Below 2.5 cm	80
		Below 5 cm	100, 110
	500 N	Recommended site	140
		Below 2.5 cm	80, 100
		Below 5 cm	110, 120
	550 N	Recommended site	120, 140
		Below 2.5 cm	80, 100
		Below 5 cm	110
Old	450 N	Recommended site	110
		Below 2.5 cm	120, 140
		Below 5 cm	80, 100
	500 N	Recommended site	100, 110
		Below 2.5 cm	120, 140
		Below 5 cm	80
	550 N	Recommended site	110, 120
		Below 2.5 cm	140
		Below 5 cm	80, 100
